# Are All Colloids Same? How to Select the Right Colloid?

**Published:** 2009-10

**Authors:** Sukanya Mitra, Purva Khandelwal

**Affiliations:** 1 Associate Professor, Department of Anaesthesia & IntensiveCare, Government Medical College & Hospital, Chandigarh; 2 Junior Resident, Department of Anaesthesia & Intensive Care, Government Medical College & Hospital, Chandigarh

**Keywords:** Colloids, Albumin, Dextran, Gelatin, Hydroxyethyl starch, Tetrastarch

## Abstract

**Summary:**

The administration of intravenous fluids is one of the most common and universal interventions in medicine. Colloids are an alternative to the frequently used crystalloids, with highly variable use depending on a myriad of clinical variables. A colloid is defined as a high molecular weight (MW) substance that largely remains in the intravascular compartment, thereby generating an oncotic pressure. Colloids are considered to have a greater intravascular persistence when compared to crystalloids. All colloids, however, are clearly not the same. Differences in the physicochemical properties, pharmacokinetics and safety profile exist amongst various colloids. This review explores the different types of colloids, with their properties and usefulness as well as adverse effects. While all the available colloids are reviewed briefly (e.g., albumin, gelatin, dextran) with respect to their pharmacology, indications, advantages and disadvantages, particular emphasis is laid on the hydroxyethyl starches (HES) because of their rising prominence. It is shown that HES differ widely in their physicochemical and pharmacokinetic properties, composition, usefulness, and especially in their adverse effect profiles. The third generation HES (tetrastarches), in particular, seem to offer a unique combination of safety and efficacy. Several issues related to this are discussed in detail. This review of the available clinical data demonstrates that HES should not be regarded as one homogenous group, and data for one product should not be automatically extrapolated to another. Thus, among the synthetic colloids, the tetrastarches appear to offer the best currently available compromise between efficacy, safety profile, and cost. They also appear to be the best suited for use in the intensive care setting. Finally, balanced (rather than saline-based) HES solutions appear promising as a plasma-adapted volume replacement strategy and may further refine the ongoing quest of finding the ideal fluid therapy.

## Introduction

The administration of intravenous fluids is one of the most common and universal interventions in medicine. Crystalloid solutions are the most frequently chosen, by far, with normal saline (NS) and lactated Ringer's (LR) both being the most frequent choices. Colloids are an alternative to crystalloids, with highly variable use depending on a myriad of clinical variables.

Clinically available colloids have generally exhibited similar effectiveness in maintaining colloid oncotic pressure. Thus, colloids have been viewed as a class of essentially interchangeable fluids and selection of colloids has commonly been based on cost and convenience. But, are all colloids same? Differences in the physicalproperties, pharmacokinetics, pharmocodynamics and safety profile exist amongst various colloids − a review of these can help us to choose the right colloid in different clinical scenarios.

## What is a colloid?

A colloid is defined as a high molecular weight (MW) substance that largely remains in the intravascular compartment, thereby generating an oncotic pressure. Colloids are considered to have a greater intravascular persistence when compared to crystalloids. This property is lost, however, when capillary membranes are altered in a diseased state.

Colloids are of two types:^1^–^4^

- Natural, i.e., human albumin
- Artificial, i.e., gelatin and dextran solutions, hydroxyethyl starches (HES).

## How do various colloids differ in their properties?

Colloids have certain general characteristics which determine their behavior in the intravascular compartment. These, along with some other characteristics of the available colloids, are shown in Table 1.

**Table 1:** Characteristics of some available colloids.

Product (Brand name)	Conc. (%)	Oncotic pressure (mmHg)	Initial volume expansion (%)	Persistence in the body (days)	Maximal daily dose (kg^−1^)
Albumin	4	20–29	80		
Albumin	25	100–120	200–400		
Dextran 70 (Macrodex)	6	56–68	120	28–42	1.5 g
Dextran 40 (Rheomacrodex)	10	168–191	200	6	1.5 g
Fluid gelatin (Geloplasma)	3	26–29	70	2–7	
Urea-linked gelatin (Haemaccel)	3, 5	25–29	70–80	2–7	
HES 670/0.75 (Hextend)	6	25–30	100		20 mL
HES 450/0.7/5 (Hetastarch)	6	25–30	100	5–6	20 mL
HES 260/0.45 (Pentastarch)	10	55–60	100–150	5–6	33 mL
HES 200/0.62/10 (Elohes)	6	25–30	110	6–7	20 mL
HES 200/0.5/6 (Hesteril)	6	30–37	100	3–4	33 mL
HES 200/0.5/6 (Lomol)	10	59–82	145	3–4	20 mL
HES 130/0.4/9 (Tetrastarch, Voluven)	6	36	130	2–3	50 mL
HES 70/0.5/3	6		100	1–2	20 mL

HES: hydroxyethyl starches. The first number appearing after HES refers to the molecular weight of the product in kilodaltons, the second number is its molar substitution ratio and the third one is the C2/C6 ratio (see text for explanations). Not all these products are available in India or with the same brand name.

- **Molecular weight (MW):** Two molecular weights are quoted for colloid solutions:
- Mw: Weight average molecular weight
- Mn: Number average molecular weight

The Mw determines the viscosity and Mn indicates the oncotic pressure. Albumin is said to be monodisperse because all molecules have the same molecular weight (so Mw = Mn).Artificial colloids are all polydisperse with molecules of a range of molecular weights.^3^

- **Osmolality and oncotic pressure:** Almost all colloid solutions have a normal osmolality. The oncocity of the solution will influence the vascular expansion. The higher the oncotic pressure, the greater the initial volume expansion.^3^
- **Plasma half-life:** The plasma half-life of a colloid depends on its MW, the elimination route, and, the involved organ function (mainly eliminated by the renal route). Half-lives of colloids vary greatly.
- **Plasma volume expansion:** The degree of volume expansion is mainly determined by the MW, whereas the intravascular persistence is determined by the elimination of the colloid. When compared to crystalloids, colloids induce a greater plasma volume expansion for the same administered volume. The duration of volume expansion varies, however, among the different colloids. Gelatins have the shortest duration of volume expansion.
- **Acid-base composition:** Albumin and gelatin solutions have physiological pH, while other solutions tend to have acidic pH.
- **Electrolyte content:** The sodium concentration is low in “salt-poor albumin”. However, the sodium content of other commercially available colloid solutions is similar to that of crystalloid solutions, while the potassium concentration differs. Urea-linked gelatin solutions contain a small, but not negligible, concentration of potassium. Calcium, similarly, is also present in the gelatin solutions.

## What are the various colloids available?

## Human albumin solution

Albumin is the principal natural colloid comprising 50 to 60% of all plasma proteins. It contributes to 80% of the normal oncotic pressure in health. Albumin consists of a single polypeptide chain of 585 amino acids with a molecular weight of 69,000 Dalton.^1^,^3^,^5^

## Metabolism

Albumin is synthesized only in the liver and has a half-life of approximately 20 days. After synthesis albumin is not stored but secreted into the blood stream with 42% remaining in the intravascular compartment^1^. When administered, two phases are observed. The first depends on the transcapillary exchange rate that corresponds to the passage of albumin from the intravascular to the extravascular compartments which occurs with the help of a transporter albondin.^3^,^5^,^6^ The second phase is a function of the fractional degradation rate.

## Degree of volume expansion:

5% solution is isooncotic and leads to 80% initial volume expansion whereas 25% solution is hyperoncotic and leads to 200 - 400% increase in volume within 30 minutes. The effect persists for 16 - 24 h.^2^

## Indications:

a. Emergency treatment of shock specially due to the loss of plasma^1^

b. Acute management of burns^1^

c. Fluid resuscitation in intensive care^5^,^7^

d. Clinical situations of hypo-albumineamia

i. Following paracentesis^8^

ii. Patients with liver cirrhosis (For extracorporeal albumin dialysis (ECAD))^8^–^10^

iii. After liver transplantation^9^

e. Spontaneous bacterial peritonitis^10^

f. Acute lung injury^11^

## Advantages:

1. Natural colloid: As albumin is a natural colloid it is associated with lesser side-effects like pruritus, anaphylactoid reactions and coagulation abnormalities compared to synthetic colloids.^12^
2. Degree of volume expansion: 25%Albumin has a greater degree of volume expansion as compared to rest of colloids. 5% albumin solution has a similar degree of volume expansion as compared to hetastarch but greater than gelatins and dextrans.
3. Other benefits: Albumin acts a principal binding protein of endogenous and exogenous substances. It also possesses antioxidant and scavenging effects. Albumin being negatively charged protein contributes to the formation of normal anion gap, influencing the acid-base status.^3^,^13^,^14^

## Disadvantages:

1. Cost effectiveness: Albumin is expensive as compared to synthetic colloids.
2. Volume overload: In septic shock the release of inflammatory mediators has been implicated in increasing the ‘leakiness’ of the vascular endothelium. The administration of exogenous albumin may compound the problem by adding to the interstitial oedema.^15^

## Dextran

Dextrans are highly branched polysaccharide molecules which are available for use as an artificial colloid. They are produced by synthesis using the bacterial enzyme dextran sucrase from the bacterium *Leuconostoc mesenteroides* (B512 strain) which is growing in a sucrose medium.^16^,^17^

## Physicochemical properties:

Two dextran solutions are now most widely used, a 6% solution with an average molecular weight of 70,000 (dextran 70) and a 10% solution with an average weight of 40,000 (dextran 40, low-molecular-weight dextran).^17^

## Metabolism & Excretion

Kidneys primarily excrete dextran solutions. Smaller molecules (14000–18000 kDa) are excreted in 15minutes, whereas larger molecules stay in circulation for several days. Up to 40% of dextran-40 and 70% of dextran-70 remain in circulation at 12 h.^2^,^16^,^17^

## Degree of volume expansion:

Both dextran-40 and dextran-70 lead to a higher volume expansion as compared to HES and 5% albumin. The duration lasts for 6–12 hours.^2^

## Indications:

- Dextran-40 is used mainly to improve micro-circulatory flow in microsurgical re-implantations.
- Extracorporeal circulation: It has been used in extracorporeal circulation during cardio-pulmonary bypass.

## Advantages:

1. Volume expansion: Dextrans leads to 100–150% increase in intravascular volume.^2^
2. Microcirculation: Dextran 40 helps in improving microcirculatory flow by two mechanisms, i.e., by decreasing the viscosity of blood by haemodilution and by inhibiting erythrocytic aggregation.

## Disadvantages:

1. **Anaphylactic reactions:** Dextrans cause more severe anaphylactic reactions than the gelatins or the starches. The reactions are due to dextran reactive antibodies which trigger the release of vasoactive mediators. Incidence of reactions can be reduced by pre-treatment with a hapten (Dextran 1).^1^,^12^ ^18^
2. **Coagulation abnormalities:** Dextrans lead to decreased platelet adhesiveness, decreased factor VIII, increased fibrinolysis and coating of endothelium is decreased. Larger doses of dextran have been associated with significant bleeding complications.^1^,^2^,^4^,^12^
3. **Interference with cross-match:** Dextrans coat the surface of red blood cells and can interfere with the ability to cross-match blood. Dextrans also increase erythrocyte sedimentation rate.^19^
4. **Precipitation of acute renal failure**: A possible mechanism for this is the accumulation of the dextran molecules in the renal tubules causing tubular plugging. Renal failure following dextran use is more often reported when renal perfusion is reduced or when preexisting renal damage is present.^2^,^3^,^12^,^20^,^21^

## Gelatins

Gelatin is the name given to the proteins formed when the connective tissues of animals are boiled. They have the property of dissolving in hot water and forming a jelly when cooled. Gelatin is thus a large molecular weight protein formed from hydrolysis of collagen.^2^,^3^,^22^ Gelatin solutions were first used as colloids in man in 1915. The early solutions had a high molecular weight (about 100,000). This had the advantage of a significant oncotic effect but the disadvantages of a high viscosity and a tendency to gel and solidify if stored at low temperatures.^22^

Several *modified gelatin* products are now available; they have been collectively called the New-generation Gelatins. There are 3 types of gelatin solutions currently in use in the world:

- Succinylated or modified fluid gelatins (e.g., Gelofusine, Plasmagel, Plasmion)
- Urea-crosslinked gelatins (e.g., Polygeline)
- Oxypolygelatins (e.g., Gelifundol)

Polygeline (‘Haemaccel’, Hoechst) is produced by the action of alkali and then boiling water (thermal degradation) on collagen from cattle bones. The resultant polypeptides (MW 12,000–15,000) are urea-crosslinked using hexamethyl di-isocyanate. The branching of the molecules lowers the gel melting point. The MW ranges from 5,000 to 50,000 with a weight-average MW of 35,000 and a number-average MW of 24,500.^3^,^22^

## Physiochemical properties:

Both succinylated gelatin and polygeline are supplied as preservative-free, sterile solutions in sodium chloride. Polygeline is supplied as a 3.5% solution with electrolytes (Na^+^ 145, K^+^ 5.1, Ca^++^ 6.25 & Cl ^−^ 145 mmol/l). As polygeline contains calcium ions it can be lead to increase in serum calcium concentration following large volume resuscitation. Polygeline also contains potassium ions: beneficial to those patients who are hypokalaemic. It is sterile, pyrogen free, contains no preservatives and has a recommended shelf-life of 3 years when stored at temperatures less than 30°C. Succinylated gelatin is supplied as 4% solution with electrolytes ((Na^+^ 154, K^+^ 0.4, Ca^++^ 0.4 and Cl ^−^120 mmol/l).As it contains low chloride it is helpful for fluid resuscitation in patients with hyperchloremic acidosis. Also succinylated gelatins are compatible with blood transfusions due to low calcium content.^22^

## Metabolism:

It is rapidly excreted by the kidney. Following infusion, its peak plasma concentration falls by half in 2.5 hours. Distribution (as a percent of total dose administered) by 24 hours is 71% in the urine, 16% extravascular and 13% in plasma. The amount metabolized is low: perhaps 3%.^2^,^3^,^22^

## Degree of volume expansion:

Gelatins lead to 70 to 80% of volume expansion. But duration of action is shorter in comparison to both albumin and starches.^3^,^4^,^22^

## Indications:

- Hypovolemia due to acute blood loss.
- Acute normovolaemic haemodilution.^23^
- Extracorporeal circulation – cardiopulmonary by-pass.^24^
- Volume pre-loading prior to regional anaesthesia.^25^

## Advantages:

1. Cost effective: It is cheaper as compared to albumin and other synthetic colloids.
2. No limit of infusion: Gelatins do not have any upper limit of volume that can be infused as compared to both starches and dextrans.
3. No effect of renal impairment: Gelatins are readily excreted by glomerular filtration as they are small sized molecules. Gelatins are associated with lesser renal impairment as compared to HMW HES.^12^,^22^

## Disadvantages:

1. Anaphylactoid reactions: Gelatins are associated with higher incidence of anaphylactoid reactions as compared to natural colloid albumin.^12^
2. Effect on coagulation: The effect of gelatins on coagulation is not clear. There are studies which support activation of coagulation by gelatins^4^ and there are some studies which reveal increased bleeding time, impaired platelet adhesiveness during cardiac surgery.^26^
3. Circulatory disturbance: Gelatins are associated with occurrence of circulatory dysfunction marked by increased plasma renin activity an aldosterone in patients with ascitis undergoing largevolume paracent sis.^27^

## Hydroxyethyl starches (HES)

HES are derivatives of amylopectin, which is a highly branched compound of starch. Amylopectin structurally resembles glycogen. Amylopectin is rapidly hydrolyzed with a ½ life of about 20 min. In order to make the amylopectin molecule more stable, anhydroxyethyl glucose residues are substituted with hydroxyethyl groups mainly at positions C_2_ and C_6_.^2^,^3^,^28^

The first HES product, Hespan (DuPont Pharmaceuticals,Wilmington, DE), was made available in the United States in the 1970s. Since then, further generations of HES have been developed, differing in their mean molecular weight (MW), molar substitution (MS), and C2/C6 ratio. Hydroxyethyl starches are identified by three numbers, e.g., 10% HES 200/0.5 or 6% HES 130/0.4. The first number indicates the concentration of the solution, the second represents the mean MW expressed in kiloDalton (kDa), and the third and most significant one is MS. These parameters are highly relevant to the pharmacokinetics of HES, as detailed below.

## Physiochemical Properties:

HES preparations are characterized by the following properties.^28^–^30^

**1.Concentration:** low (6%) or high (10%).

Concentration mainly influences the initial volume effect: 6% HES solutions are iso-oncotic in vivo, with 1 l replacing about 1 l of blood loss, whereas 10% solutions are hyperoncotic, with a volume effect considerably exceeding the infused volume (about 145%).

**2.Average Molecular Weight (MW):** low (≃70 kDa), medium ( 200 kDa), or high ( ≃ 450 kDa).

In common with all of the synthetic colloids, HES are polydisperse systems containing particles with a wide range of molecular mass. In polydisperse systems, the determination of particle mass or relative molecular mass gives averages, which depend on the method used as mentioned earlier (Mw and Mn). The ratio Mw/Mn gives an index of the degree of polydispersity in the system. When a polydisperse colloid is infused into the circulation, small molecules below the renal threshold (45 to 60 kDa7) are rapidly excreted, whereas the larger molecules are retained for varying periods of time depending on their size and ease of breakdown. However, osmotic effectiveness depends on the number of particles, and not the molecular size; therefore, the excretion of the smaller particles continuously reduces the osmotic effectiveness of the infused solution. This is compensated for by the continuous supply of oncotically active molecules arising from degradation of larger fragments. Mean MW of the available products ranges from over 670 kDa to 70 kDa **(Table 2).**

**Table 2:** Comparative efficacy and safety of albumin vs. HES.

COLLOID	ALBUMIN	HETASTARCH	TETRASTARCH

COST	Expensive	Cheap	Expensive
USE	Long term	Short term	Any
PERIOPERATIVE:			
COAGULATION PROFILE	No increased bleeding	Increased bleeding	No increased bleeding
RENALTRANSPLANTATION	Safe	Predisposes to acute renal failure, oliguria	No evidence of risk till date
PRE-EXISTINGIMPAIRMENT			
·RENAL	Safe	Osmotic nephrosis like lesions	No evidence of risk till date
·HEPATIC	Safe	Ascitis, accumulation	No evidence of risk till date

**3.Molar substitution (MS):** low (0.45–0.58) or high (0.62–0.70)

The degree of substitution refers to the modification of the original substance by the addition of hydroxyethyl groups. The higher the degree of molar substitution, the greater the resistance to degradation, and consequently, the longer its intravascular persistence.

HES have a varying number of hydroxyethyl residues attached to the anhydrous glucose particles within the polymer. This substitution increases the solubility of the starch in water and, to a varying degree, inhibits the rate of destruction of the starch polymer by amylase. As with MW, there are two methods for calculating the degree of substitution on the starch polymer. The first of these is termed the degree of substitution and is calculated from the number of substituted anhydroglucose residues divided by the total number of anhydroglucose residues. The second is generally referred to as the MS, which is calculated as the average number of hydroxyethyl groups reacted per anhydroglucose residue. The numbers represent the mass of the hydroxyethyl group and the anhydrous glucose residue, respectively.

MS is thus the average number of hydroxyethyl residues per glucose subunit. The figure 0.7 in the description of a HES preparation indicates that there are seven hydroxyethyl residues on average per 10 glucose subunits. Starches with this level of substitution are called hetastarches, and similar names are applied to describe other levels of substitution: hexastarch (MS 0.6), pentastarch (MS 0.5), and tetrastarch (MS 0.4).

Unsubstituted anhydroglucose units are more prone to enzymatic degradation by alpha-amylase; therefore, hydroxyethylation slows down the rate of enzymatic breakdown of the HES molecule and prolongs intravascular retention time. Thus, older generation HES products with high MS accumulate in the plasma, unlike the latest generation of tetrastarches.

**4.C2/C6 ratio:** low (<8) or high (>8).

The C2/C6 ratio refers to the site where substitution has occurred on the initial glucose molecule. The higher the C2/C6 ratio, longer the half-life and hence, longer persistence in the blood. Thus, the *pattern* of hydroxyethylation also has a significant impact on the pharmacokinetic properties, but this may not be appreciated because it does not appear in the usual product specification alongside MW and MS. Hydroxyethylation of the glucose subunits is guided predominantly towards the C2 and C6 carbon atoms. Hydroxyethyl groups at the position of the C2 atom inhibit the access of alpha-amylase to the substrate more effectively than do hydroxyethyl groups at the C6 position. Hence, HES products with high C2/C6 ratios are expected to be more slowly degraded.

## Metabolism:

Following the infusion of HES there is initially a rapid amylase-dependent breakdown and renal excretion. Plasma half life is 5 days and 90% is eliminated in 42 days.^30^ Smaller HES molecules (<50,000 to 60,000 Dalton) are eliminated rapidly by glomerular filtration. Medium sized molecules get excreted into the bile and faeces. Another fraction is taken up by the reticuloendothelial system (RES) where the starch is slowly broken down. Thus, trace amounts of the preparations can be detected for several weeks after administration.^1^,^2^,^3^

## Degree of volume expansion:

The increase in colloid osmotic pressure obtained with HES is equivalent to albumin. HES results in 100% volume expansion similar to 5% albumin. It results in greater volume expansion as compared to gelatins.^31^ Duration of volume expansion is usually 8-12 h.^2^

## Indications:

- Stabilization of systemic haemodynamics.
- Anti-inflammatory properties: HES has been shown to preserve intestinal microvascular perfusion in endotoxaemia due to their anti-inflammatoryproperties.^32^

## Advantages:

1. Cost effectiveness: HES is less expensive as compared to albumin and is associated with a comparable volume of expansion.
2. Maximum allowable volume: Maximum volume which can be transfused of medium weight HES (130 kDa) with medium degree of substitution (0.4) is 50 ml/kg. This is greater as compared to other synthetic colloids like dextrans.

## Disadvantages:

The first and second-generation HES (Hextend, Hetastarch, Pentastarch) are associated with various side-effects as follows:

1. **Coagulation:** HES administration is associated with reduction in circulating factor VIII and von Willebrand factor levels, impairment of platelet function, prolongation of partial thromboplastin time and activated partial thromboplastin time and increases bleeding complications.^2^,^4^,^12^,^29^
2. **Accumulation:** High molecular weight (HMW) HES are associated with greater degree of accumulation in interstitial spaces and reticulo-endothelial system. It gets deposited in various tissues including skin, liver, muscle, spleen, intestine, trophoblast and placental stroma. Such depositions have been associated with pruritus.^2^,^12^ ^33^
3. **Anaphylactoid Reactions:** HES is associated with higher incidence of anaphylactoid reactions as compared to other synthetic colloids as well as albumin.^12^
4. **Renal impairment**: HMW HES has been found to be associated with increased creatinine levels, oliguria, acute renal failure in patients who were critically ill with existing renal impairment.^34^,^35^ HMW HES is associated with development of osmotic nephrosis like lesions in both proximal and distal renal tubules.^35^ Cittanova et al demonstrated that the use of 6% HES 200/0.62 (2,100 ± 660 ml) in brain-dead donors resulted in impaired renal function in kidney transplant recipients.^36^ Thus, HES preparations with high molecular weight and/or high MS may have detrimental consequences for renal function. Modern HES preparations with lower Mw, lower MS (e.g., HES 130/0.4) have been shown to have no more negative influence on kidney function.
5. **Increase in amylase levels:** HES infusion is an occasional elevation of the serum amylase levels. But this has no clinical implication as such.^2^,^3^
  However, it is important to consider the data for individual products and not to extrapolate reports from one HES type to another. Clinical studies have revealed significant differences between the HES generations regarding coagulation, tissue storage, and renal function. The next section on “third-generation” HES (also known as tetrastarch) clearly brings out this important point.

## Third-generation HES: tetrastarch

The development of newer starch-based plasma volume expanders has been driven by a need to improve safety and pharmacological properties while maintaining the volume efficacy of previous HES generations.^28^,^37^ Reductions in MW and MS have led to products with shorter half-lives, improved pharmacokinetic and pharmacodynamic properties, and fewer side effects. Although earlier products were derived from amylopectin extracted from waxy maize starch, it is inaccurate to refer to HES as if they were only one homogenous product because modifications to MW and the degree and pattern of substitution result in distinct and observable differences between and within the different generations of HES. The same is true for starches of similar structure that have been derived from different source materials: waxy maize and potato. Two third-generation starches based on these two materials are currently available in various formulations. According to one study, potato and waxy maize-derived HES solutions are not bioequivalent.^38^ Therefore, findings obtained from studies using one type may not be valid for the other.

Safety profile of tetrastarches vis-à-vis earlier-generation HES

**Effects on Coagulation and Platelet Function:** A number of studies have investigated the in vitro and in vivo effects of various HES products on coagulation and platelet function. Overall, the more rapidly degradable HES products have been found to have a greatly reduced effect on the coagulation process compared to older products. The most useful evidence concerning the safety of waxy maize-derived 6% HES 130/0.4 is derived from extensive clinical studies in many types of major surgery. Although very high doses have been used, no adverse effects on coagulation have been reported compared to controls using lower doses.

A pooled analysis of prospective and random ized studies comparing 6% HES 130/0.4 with 6% HES 200/0.5 in patients undergoing major surgical procedures (n = 449) was carried out by Kozek-Langenecker et al.^39^ The authors concluded that HES 130/0.4 was associated with a significant reduction in perioperative blood loss, both estimated and calculated, and that there was a significant reduction in transfusion needs. The reduction in the volume of erythrocyte loss and in transfusion needs was in the order of one red blood cell unit for both parameters.

A meta-analysis including 73 randomized trials compared the clinical outcome in adult patients receiving colloids in the perioperative period.^40^ HES were stratified according to MS. It was found that tetrastarches were associated with a 15% reduction in blood loss compared to gelatin and pentastarches. Pentastarches were associated with larger perioperative blood loss (10%) as compared to albumin. All other clinical outcome variables were similar between groups. The evidence base for waxy maizederived HES (6% 130/0.4) is particularly strong; overall, there are more than 50 published studies reporting on the coagulation effects of waxy maize-derived HES 130/0.4, including more than 20 Phase II to IV studies. These studies confirm that, unlike earlier generation HES preparations, the tetrastarches have minimal effect on coagula tion.^28^

**Accumulation and Tissue Storage:** HES molecules with a higher in vivo MW resulting from increased MS tend to be stored in tissue before being metabolized by amylases. Due to the more rapid clearance of the latest generation of tetrastarches, it is expected that tissue accumulation and its clinical manifestations will not be observed with the same frequency as compared to older starches. The main clinical manifestation of tissue storage is HES-related pruritus, which was first reported in otologic patients who had received relatively high repeated doses of HES. The pruritus arises from long-term cutaneous storage of HES molecules, and it may last for months after exposure. The incidence appears to be related to the MS and the cumu lative infused dose, and it is resistant to treatment by glucocorticoids, antihistamines, acetaminophen, and neuroleptic drugs. By comparison, Ellger et al found no incidence of postoperative itching in any of the 40 patients undergoing elective urologic cancer surgery, although relatively high doses of waxy maize-derived HES 130/0.4 (6%) were given.^41^ In other studies of HES 130/0.4 using relatively high doses, pruritus did not seem to be a clinical problem.^42^,^43^

**Effects on Plasma Bilirubin:** Waxy maize-derived HES 130/0.4 has been extensively studied in a large number of clinical trials. None of these reports suggests that it is associated with deterioration of liver function compared to controls. However, potato-derived HES 130/0.42 is the only tetrastarch to be absolutely contraindicated in patients with severe hepatic impairment.^28^

**Effects on Renal Function:** A number of earlier reports suggest that HES products may have adverse effects on renal function.^34^–^36^ However, more recent studies using third-generation products have not reported unfavorable effects, suggesting that the lower tendency of these products to accumulate may improve their profile with regard to renal function.

An important large-scale observational study of the effects of HES administration on renal function was carried out by Sakr et al.^44^ In a retrospective analysis of data of 3147 critically ill patients included in the SOAP study (Sepsis Occurrence in Acutely Ill Patients), it was found that HES per se was not an independent risk factor for adverse effects on renal function in the 1,075 patients who received HES. Neither the use of HES nor the dose administered was associated with an increased risk of renal replacement therapy, even in the subgroup of patients with severe sepsis and septic shock (n = 822). These patients were also at particular risk for renal dysfunction because of a high incidence of cardiovascular dysfunction and preexisting renal impairment. Unfortunately, the authors did not distinguish between the types of HES preparations used; however, they did acknowledge that the use of newer HES preparations with a lower tendency to accumulate may have contributed to the favorable results.

In the considerable body of clinical data on the third generation HES 130/0.4, there have been no reports of adverse effects on renal function over and above those seen in control groups in patients who are considered to be at particular risk, such as those with previous mild to severe renal dysfunction,^45^ the elderly,^46^ and those receiving high-dose therapy.^47^

In summary, the published data on this topic suggest that there are differences between the older and newer generations of HES and that the reports of adverse effects on renal function should not be extrapolated to newer HES products. Nine clinical trials on renal function demonstrate the safety of waxy maize-derived HES 130/0.4, and two recently published trials confirm that potato-derived HES 130/0.42 has no adverse effects on renal function either.^28^,^37^

**Special Patient Groups:** Extra caution is always needed when treating high-risk groups, such as the elderly, children, and those with renal impairment. Due to a higher incidence of comorbidities and changes in lung, kidney and cardiovascular function, the **elderly** are at increased risk for impairment of renal function. The waxy maize-derived tetrastarch HES 130/0.4 has been thoroughly studied in these groups and has a well-documented safety profile. In the elderly, HES 130/0.4 has been studied in patients undergoing abdominal surgery, where it was found to be an adequate replacement for albumin or gelatin. In cardiac surgery patients, HES 130/0.4 was deemed to be as safe as gelatin, offering a more persistent volume effect and a lower risk of anaphylactoid reaction. Further studies on HES 130/0.4 have also confirmed its safety in surgery, where patients are at high risk for renal dysfunction: abdominal aortic surgery, spinal fusion surgery, and surgery for aortic aneurysm.^28^,^37^,^45^

Waxy maize-derived HES 130/0.4 is the only third generation HES with controlled clinical data in **children**. In this context, Standl et al reported that waxy maize-derived 6% HES 130/0.4 was as safe and well tolerated as albumin when used in pediatric surgery.^48^ Other studies reached similar conclusions when using 6% HES 130/0.4 and 4% albumin in pediatric cardiac surgery and spinal fusion, whereas Sumpelmann et al reported a very low level of adverse reactions with potato-derived HES 130/0.42 in a noncomparative observational study in children.^49^

**Effects on Microcirculation and Oxygenation:** There is increasing evidence that some plasma substitutes possess additional properties that have beneficial effects on organ perfusion, microcirculation, tissue oxygenation, inflammation, endothelial activation, capillary leakage, and tissue edema over and above their volume replacement effects. Hypovolaemia may initiate a cascade of pathophysiological processes, such as stimulation of the sympathoadrenergic and reninangiotensin systems that may result in inadequate tissue perfusion and decreased oxygen supply to the tissues. Ideally, therefore, fluid therapy should confer beneficial effects on microcirculation and tissue oxygenation.

Third generation HES 130/0.4 has positive effects on tissue oxygenation and microcirculation in patients undergoing major abdominal surgery.^50^ Intravascular volume replacement with a 6% solution improved tissue oxygenation compared with a crystalloid-based volume replacement strategy using lactated Ringer's titrated to similar hemodynamic endpoints. The tetrastarch was also found to produce a greater and earlier increase of tissue oxygen tension as compared to two pentastarch solutions (6% HES 70/0.5 and 6% HES 200/0.5) when administered to volunteers and a more pronounced and earlier increase of skeletal muscle oxygen tension. Lang et al^50^ attribute these beneficial effects of tetrastarches to improved microperfusion and reduced endothelial swelling; crystalloids mostly distribute in the interstitium, causing endothelial tissue swelling and reduced capillary perfusion. Neff et al^51^ suggest that HES with lower MS may decrease erythrocyte aggregation, thereby reducing low-shear viscosity of the blood. However, more studies are needed to investigate this issue more thoroughly.^28^

## Colloid vs. crystalloid for volume resuscitation in the critically ill

So, are both colloids and crystalloids safe and effective means of intravenous fluid resuscitation? Although this has been the most common assumption over the past 60 years, it may not be true. The safety of colloids was first questioned by a rudimentary meta-analysis performed by Velanovich^52^ in 1989. Since that time, there have been a number of other more elegant systematic reviews that have similarly questioned the safety of colloids. The first of these were published in BMJ in 1998 in which one systematic review questioned the safety of colloids in general^53^ and another questioned specifically the safety of albumin.^7^ Both of these meta-analyses suggested that there was a small but statistically significant increase in the risk of death for patients who received colloids over crystalloids. Since that time, there has been a rigorous and more focused meta-analysis, including an assessment of potential morbid complications of colloid use, which found no difference in outcome among patients treated with colloids or crystalloids.^54^

However, the questions regarding the safety of colloids remained in clinicians’ minds and circulated in the literature. Based on these concerns, the Australia and New Zealand Intensive Care Society's Clinical Trials Group (ANZICS-CTG) designed and conducted one of the largest critical care trials in history.^55^ The SAFE (Saline versus Albumin Fluid Evaluation) trial randomized 7000 critically ill patients requiring fluid resuscitation to receive isooncotic albumin or isotonic crystalloid. In this study, there was no overall difference in outcome according to whether patients received colloids or crystalloids (relative risk for death with colloid use = 0.99, 95% confidence interval 0.91–1.09, *P*=0.87).

## Clinical efficacy of different colloids

Important effects of prophylactic or therapeutic administration of HES colloids are the maintenance and rapid restoration of intravascular volume. Besides these effects on macrocirculation, effects on microcirculation and tissue oxygenation are important for the preservation of organ function. HES 130/0.4 (6%) was found superior regarding tissue oxygenation when compared with crystalloids in major abdominal surgery,^50^ and provided a larger and faster increase of tissue oxygen tension when compared with other HES solutions after infusion in volunteers.^56^

HES differs from other pharmaceutical active ingredients like small molecules or albumin because of its polydispersity and because of changes in molecular weight from in vitro to in vivo situations. Pharmacokinetic parameters such as half-lives cannot be defined rigorously. HES clearances and residual HES concentrations after 24 hours, however, clearly depend on the molar substitution and the C2/C6 ratio, whereas the initial mean molecular weight in the bottle is of secondary importance. In the case of HES products, plasma concentration half-lives should not erroneously be interpreted as efficacy half-lives. Intravascular volume is known to be regulated by a number of mechanisms including the colloid osmotic pressure which is raised by infusion of colloid solutions. Counter-regulatory mechanisms after plasma volume expansion have to be taken into account. Therefore, the extent and duration of the volume effects induced by the infusion solution, besides the type of infusion, also highly depend on the individual patient's condition, blood loss status, infusion dose and speed. A longer plasma persistence of HES was initially regarded as favorable as this was thought to result in a prolonged volume effect. A large number of studies, including initial uncontrolled observations till recent double-blind randomized controlled trials clearly demonstrate that this belief was not justified.^57^–^59^ For example, a recent double-blind study by Gandhi et al^60^ performed in the USA investigated the efficacy and safety of HES 130/0.4 and HES 670/0.75 in patients undergoing major orthopaedic surgery. In-fusion of the colloids was guided by a predefined algorithm taking central venous pressure and arterial blood pressure into account. The results clearly showed that both colloids were equally effective in the stabilization of haemodynamics. Volume of infused crystalloids was similar in both groups.

Current guidelines on initial haemodynamic stabilization in shock states suggest infusion of either natural or artificial colloids or crystalloids. However, as the volume of distribution is much larger for crystalloids than for colloids, resuscitation with crystalloids alone requires more fluid and results in more oedema, and may thus be inferior to combination therapy with colloids. According to a latest critical review of the use of various colloids in intensive care medicine,^61^ dextrans appear to have the most unfavourable risk/benefit ratio among the currently available synthetic colloids due to their relevant anaphylactoid potential, risk of renal failure and, particularly, their major impact on haemostasis. The effects of gelatin on kidney function are currently unclear, but potential disadvantages of gelatin include a high anaphylactoid potential and a limited volume effect compared with dextrans and HESs. Modern HES preparations have the lowest risk of anaphylactic reactions among the synthetic colloids. Older HES preparations (hetastarch, hexastarch and pentastarch) have repeatedly been reported to impair renal function and haemostasis, especially when the dose limit provided by the manufacturer is exceeded, but no such effects have been reported to date for modern tetrastarches compared with gelatin and albumin. However, no large-scale clinical studies have investigated the impact of tetrastarches on the incidence of renal failure in critically ill patients. When considering the efficacy and risk/ benefit profile of synthetic colloids, modern tetrastarches appear to be most suitable for intensive care medicine, given their high volume effect, low anaphylactic potential and predictable pharmacokinetics. However, the impact of tetrastarch solutions on mortality and renal function in septic patients has not been fully determined, and further comparison with crystalloids in prospective, randomized studies is required.^61^

## Saline versus balanced HES

Conventional HES solutions consist of saline with abnormally high concentrations of sodium (154 mmol/l) and chloride (154 mmol/l). A total balanced volume replacement strategy is a new concept for correcting hypovolemia. To fulfill this concept, balanced colloids, for example, balanced hydroxyethyl starch (HES) solutions, are necessary in addition to balanced crystalloids. In animal as well as in human studies, the use of HES dissolved in a plasma-adapted solution showed beneficial effects on acid—base status compared with conventional HES dissolved in saline (reviewed recently by Boldt^62^). As the base excess is an important surrogate marker for identifying patients with malperfused tissues, infusion of considerable amounts of unbalanced HES solutions producing low base excess would possibly result in inappropriate clinical interventions. Balancing the HES preparation was associated with significantly fewer alterations in coagulation; dilution of blood with balanced HES showed significantly fewer negative effects on thrombelastography and platelet aggregation than conventional HES. Whether modulation of the acid—base status by a balanced volume replacement strategy would beneficially influence organ function, morbidity or even mortality in the critically ill must be evaluated in large controlled future studies. At present, arguing against a total balanced volume replacement strategy appears to be difficult. Balanced HES solutions complete the idea of a plasma-adapted volume replacement strategy and may add another piece to the puzzle of finding the ideal fluid therapy for treating the hypovolemic patient.^62^

Making a choice in regards to colloids requires the clinician to have a thorough knowledge of the different properties and side-effects of various available preparations. Laboratory, animal, and clinical studies all demonstrate that there are clear physicochemical and pharmacokinetic differences between the generations of HES, mainly resulting from modifications to the MS and the pattern of substitution. Both of these result in differences in the in vivo MW as well as plasma and tissue persistence. Apparently small variations in MS have significant effects on the coagulation system and renal function. Notably, the third generation of tetrastarches shows a significantly improved safety profile without any loss of volume effect compared to first-and second generation HES preparations. The increased safety margin of HES 130/0.4 was recognized by European regulatory authorities when the maximum daily dose was increased to 50 mL/kg which is the highest dose limit for any HES type approved for human use so far. Variation in the source material for HES also produces measurable pharmacokinetic differences in the end product. This review of the available clinical data demonstrates that HES should not be regarded as one homogenous group, and data for one product should not be extrapolated to another.

The development of new HES molecules was guided towards faster and more complete elimination. For the latest generation HES (molar substitution 0.4), clearances more than 23 times higher than for first-generation hetastarch and almost five times higher than for second generation pentastarch have been shown. Consequently, tissue storage could be greatly reduced, and plasma accumulation is virtually absent after multiple dosing. Nevertheless, as proven by several well-de-signed double-blind trials, volume efficacy of HES 130/0.4 is equivalent to HES 200/0.5 as well as to HES 670/0.75. The prior belief that prolonged intravascular retention is associated with a prolonged volume effect was not justified, as even slowly metabolizable HES types do not cause considerable volume effects 24 hours after the last administration.

Complex issues exist when discussing intravenous fluids. A more logical approach is to select the type of fluid that is best designed to treat a specific problem. Crystalloid fluids should be used in patients with dehydration, i.e., with loss of both interstitial and intravascular fluid. Colloid fluids are designed to stay in the intravascular space. Currently available evidence suggests that perioperative volume therapy should aim at optimizing plasma volume against dynamic endpoints using colloid. Crystalloid use should be limited to replacement of deficits and ongoing clear-fluid losses. Among the synthetic colloids, the tetrastarches appear to offer the best currently available compromise between the cost of products such as albumin and safety profile. Finally, balanced HES solutions appear promising as a plasma-adapted volume replacement strategy and may further refine our ongoing quest of finding the ideal fluid therapy.
